# Preliminary Outcome of a Novel Edge-to-Edge Closure Device to Manage Mitral Regurgitation in Dogs

**DOI:** 10.3389/fvets.2020.597879

**Published:** 2020-12-17

**Authors:** Bo Liu, Stacey B. Leach, Wenzhi Pan, Fangyu Zheng, Liujun Jia, Xueying Zhou, Jing Li

**Affiliations:** ^1^College of Veterinary Medicine, China Agricultural University, Beijing, China; ^2^Department of Veterinary Medicine and Surgery, College of Veterinary Medicine, University of Missouri, Columbia, MO, United States; ^3^Department of Cardiology, Shanghai Institute of Cardiovascular Disease, Zhongshan Hospital, Fudan University, Shanghai, China; ^4^Beijing Key Laboratory of Pre-Clinic Research and Evaluation for Cardiovascular Implant Materials, Fuwai Hospital National Cardiovascular Centre, Peking Union Medical College and Chinese Academy of Medical Sciences, Beijing, China

**Keywords:** canine, mitral valve, myxomatous degeneration, transapical, valve surgery

## Abstract

**Background:** Veterinary management of mitral valve regurgitation due to mxyomatous valve disease in dogs is limited to medical treatments, which only postpones the onset of congestive heart failure or alleviates clinical symptoms. Most surgical procedures to manage this condition in humans require cardiopulmonary bypass and have a high risk of complications.

**Animals:** Eight dogs with naturally occurring mitral valve regurgitation.

**Methods:** Prospective observational study. All dogs were treated with a novel edge-to-edge transcatheter device named ValveClamp. The total surgical procedural time and total catheterization time were recorded. Echocardiographic variables measured pre- and post-procedure were compared using Wilcoxin-signed rank test with a *P* < 0.05 considered significant. Data were expressed as median and interquartile range and absolute numbers and percentages.

**Results:** The procedural success rate was 100% and all the dogs survived without complications. The median (interquartile range) total surgical procedural time was 86.5 (76–96.2) minutes and catheterization time was 23.5 (22–33.8) minutes. Echocardiography revealed a significant reduction in mitral regurgitation severity in all dogs following the procedure based on both a reduced mitral regurgitant maximum jet area (*P* = 0.012) and a reduced mitral regurgitant maximum jet area to left atrial area (*P* = 0.018).

**Conclusion:** The ValveClamp device is effective at reducing the severity of mitral regurgitation in dogs with naturally occurring myxomatous valve disease.

## Introduction

Myxomatous mitral valve disease (MMVD) is the most common cardiac disease in dogs, primarily affecting small breeds (1). The standard care for this disease is primarily medical therapy, while surgical treatment is performed in a minority of dogs (2). Medical therapy postpones the onset of congestive heart failure (CHF), and also increases the survival time once CHF develops (3–6). However, medical treatment has minimal effect on the degree of mitral regurgitation (MR) and does not reverse the severity of the disease (7, 8). Thus, the long-term prognosis is poor despite medical intervention. In human medicine, one of the standard treatments for MMVD is surgical mitral valve repair via thoracic surgery. Given the risk associated with surgical repair and cardiopulmonary bypass, novel less invasive catheterization techniques are emerging (9–11). Some surgical techniques have been applied clinically and experimentally in small animals (12–15).

Medical management is currently the only treatment option in China for dogs with MMVD. We have previously reported a novel trans-catheter edge-to-edge mitral valve device named ValveClamp (Hongyu Medical Technology, Shanghai, China) that was developed using a swine model for mitral valve regurgitation, and the first human case was also recently documented (16, 17). The aim of the current study was to evaluate the performance of this device in the treatment of clinical cases of dogs with confirmed mitral regurgitation secondary to MMVD.

## Materials and Methods

This study was approved by the animal welfare and ethics committee of China Agricultural University and the State Key Laboratory of Cardiovascular Diseases Ethics Committee (AW28060202-2), and was performed with written owner consent.

All procedures were conducted at the Clinical Medicine Institute, Zhongshan Hospital, Shanghai. Dogs were selected for the study based on the presence of a left apical systolic heart murmur heard on auscultation and mitral regurgitation confirmed by echocardiography. The intensity of the murmur was graded using the conventional grade I–VI scale as previously described (18). The dogs were otherwise healthy based on the physical examination, complete blood count, and urinalysis. An additional inclusion criterion was that the dogs could not be receiving any cardiac specific pharmacotherapy. All dogs had an echocardiogram performed at baseline (pre-procedure) and immediately after the clamp placement. Echocardiography was performed using either the Philips CX50 (*n* = 3) or Epiq 7C (*n* = 5) ultrasound machines with transducers ranging from 1 to 7 MHz (Philips Medical Systems, Andover, MA, USA). The pre-procedural echocardiogram was used to determine confirm the presence of mitral regurgitation, measure the left atrial to aortic root ratio (LA:AO), and determine the stage of MMVD using the previously reported ACVIM staging scheme (1). Semi-quantification of mitral regurgitation severity was performed at baseline and post-operatively using color-flow Doppler to determine the ratio of the mitral regurgitant maximum jet area (MRA) to left atrial area (LAA), with regurgitation considered trace if the MRA/LAA was <5%, mild if >5% and <20%, moderate if >20% and <50%, and severe if >50%. This method has been previously validated in dogs and has been shown to correlate well Doppler derived regurgitant volume and effective regurgitant orifice area (19–21). Other specific echocardiographic measurements evaluated at both time points included the maximum transvalvular diastolic pressure gradient, mean transvalvular diastolic pressure gradient, and left atrial anterior–posterior diameter.

The ValveClamp mitral valve repair system used in this study was identical to that previously developed in pigs (16). The procedure was performed under general anesthesia with mechanical ventilation. The dogs were pre-medicated with butorphanol (0.2 mg/kg IV) and induced with propofol (2–4 mg/kg IV titrated to effect) before intubation. General anesthesia was maintained with isoflurane (1–2.5%) with 100% oxygen. Heparinized saline (100 U/kg IV, then 10–25 U/kg/hr IV CRI) was given to the patients during surgery to reduce the risk of clotting, keeping the activated clotting time between 300 and 600 s. Dogs were instrumented for continuous monitoring of SpO_2_, ECG, end-tidal CO_2_, and non-invasive blood pressure. The total surgical procedural time (from incision to closure) and the total catheter procedural time (from pericardial puncture to suturing) was recorded.

The detailed procedure for using the system has been described previously (16). In short, the apex of the heart was exposed via a 5–8-cm intercostal minithoracotomy incision at the left 5th intercostal space near the sternum. A purse-string suture of 2-0 polypropylene monofilament (Jinghuan, Shanghai, China) was placed in the cardiac apex, which encircled a stab incision through which a 14F sheath (APT Medical Tech, Hunan, China) was passed into the left ventricle. The ValveClamp mitral valve repair system was passed through the sheath and used to guide the sheath across the mitral valve and into the left atrium. The distal part of the clamp was released into the left atrium and positioned under transesophageal or direct epicardial echocardiographic guidance. The catheter was then partially withdrawn so that the proximal half of the clamp was released into the left ventricle. The two halves of the clamp were then tightened, capturing the mitral leaflets between them. If the valves were not sufficiently well-trapped the clamp arms were released and the process was repeated. Once the valve flaps were adequately secured, the guide was released and removed, leaving the clamp *in situ*. The size of the clamp was selected based on the mitral valve annulus diameter of each animal. Type I (14 mm) clamps were selected for dogs with mitral valve annulus diameter <20 mm, and Type II (16 mm) clamps for 20–30 mm. Heparinized saline was tapered off during recovery. Dogs were prescribed meloxicam (0.2 mg/kg PO once followed by 0.1 mg/kg PO q 24 h) and tramadol (4 mg/kg PO TID) for 2 weeks for post-operative pain relief. Dogs were medicated with clopidogrel (2 mg/kg PO BID) upon recovery and for 30 days after surgery.

### Statistical Analysis

Given the small sample size, the data was assumed to have a non-normal distribution. All results Continuous variables were expressed as median and interquartile range (IQR) unless otherwise indicated. Categorical variables were expressed as absolute numbers and percentages. Comparisons of variables (MRA, MRA/LAA, maximum diastolic transvalvular gradient, mean diastolic transvalvular gradient, left atrial anterior–posterior diameter, and mitral annulus diameter) between pre- and post-procedure evaluations were compared with Wilcoxon signed-rank test. Statistical analysis was performed using a commercial software program (SPSS, version 26.0, Armonk, NY, USA). A *P* < 0.05 was considered significant.

## Results

Between March 2020 and April 2020, eight adult dogs (3 miniature poodles, 2 Pomeranians, and 3 mix-breeds; 5 males and 3 females) were enrolled in this study. Median (IQR) age was 6.5 (5.8–7.2) years and median (IQR) weight was 4.5 (3.9–4.7) kg. The murmur was graded II/VI in 1/8 dogs (12.5%), III/VI in 5/8 dog (62.5%), IV/VI in 2/8 dog (25%). The median LA:AO was 1.28 (range 1.13–1.57) indicating all dogs had stage B1 MMVD.

Based on the mitral annular diameter, a Type I clamp was used in all dogs. The procedure was successfully completed in every dog, with all dogs surviving surgery and recovering from anesthesia without complications. All surgical incisions healed without complications. The median (IQR) total procedure time from thoracic opening to closure was 86.5 (76–96.2) minutes and the median (IQR) catheter procedure time from pericardial puncturing to pericardial suturing was 23.5 (22–33.8) minutes. At recheck evaluation 3 months post-procedure, an audible murmur could not be heard in any dog. Recheck echocardiography revealed no detectable mitral regurgitation in 7/8 (87.5%) dogs and trace regurgitation in only 1/8 (12.5%) dogs, with no visible thrombus detected in any dog. No major complications have been reported in any of the dogs to date, with a mean follow-up time of 124 days since surgery (range from 100 to 141 days).

**Figure 1:**
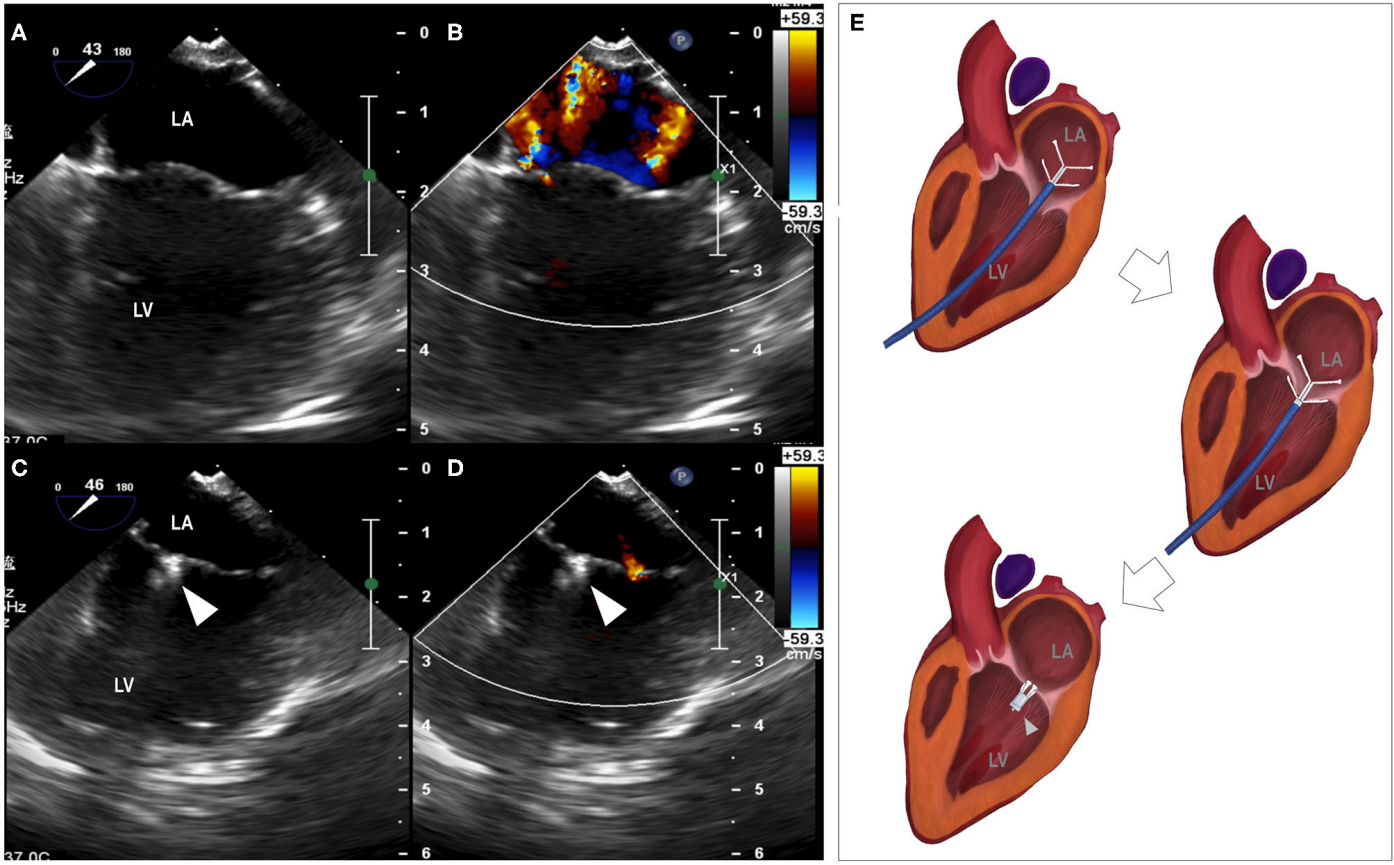
Reduction of mitral regurgitation. **(A)** median longitudinal view of left atrium and left ventricle before the procedure, **(B)** mitral regurgitation revealed by color Doppler, **(C)** median longitudinal view of left atrium and left ventricle with the placement of the clamp, **(D)** minimal mitral regurgitation detected by color Doppler. **(E)** Animation of the clamping procedure. LA, left atrium; LV, left ventricle; arrowhead, the clamp.

Descriptive data for hemodynamic, cardiac dimensional and functional metrics are summarized in Table 1. A significant reduction in the severity of mitral regurgitation was observed based on reductions in both the MRA (*P* = 0.018) and MRA/LAA (*P* = 0.012) following the procedure. The reduction of mitral regurgitation occurred instantly once the clamp was applied, as demonstrated by echocardiographic monitoring during the procedure (Figure 1). Only trace to mild mitral regurgitation was present post-procedure. The size of the left atrium and mitral annulus and the transvalvular diastolic pressure gradients all remained unchanged.

**Table 1:** Hemodynamic, cardiac-size and mitral valve functional changes after the ValveClamp procedure.

	**Pre-procedure median (IQR)**	**Post-procedure median (IQR)**	***P*-value**
MRA/LAA (%)	57 (38–59)	0 (0–1)	0.018
MRA (mm^2^)	1.3 (0.88–1.74)	0 (0–0.08)	0.012
Maximum transvalvular diastolic pressure gradient (mmHg)	1 (1–2)	1 (1–3)	0.194
Mean transvalvular diastolic pressure gradient (mmHg)	1 (0–1)	0 (0–1)	0.317
LA anterior–posterior diameter (mm)	16.1 (13.2–19.4)	12.7 (12.1–15.2)	0.058
LAA (mm^2^)	3.4 (2.1–4.3)	2.6 (2–3.3)	0.063
Mitral annulus diameter (mm)	15.8 (15.4–16.3)	13.1 (11.7–14.4)	0.068

## Discussion

This study shows that the ValveClamp device can be successfully used in dogs and may be an effective clinical treatment for dogs suffering from MMVD. Significant reduction in mitral regurgitation was observed in all dogs after the procedure by measuring the MRA and the MRA/LAA. Additionally, the unchanged transvalvular diastolic pressure gradients indicate that mitral stenosis did not occur. The ValveClamp device is functionally similar to the expanded polytetrafluoroethylene (ePTFE) chordal implantation device and neither technique requires cardiopulmonary bypass, which is required in procedures of mitral valve replacement and mitral annuloplasty (12–14, 22). The ePTFE chordal implantation technique involves transapical delivery of ePTFE artificial cords that are anchored to the affected valve leaflets and secured to an ePTFE pledget on the epicardium (15). The ePTFE chordal implantation procedure has been previously evaluated in a pilot study in dogs, though both intra-operative death and post-operative complications were noted (15). The procedure to implant the ValveClamp device is technically more straightforward and appears to be prone to fewer intraoperative complications. The 100% survival rate and rapid recovery of dogs following this procedure are highly encouraging. However, the post-operative period for all dogs presented here is still relatively short and longer follow-up is required to demonstrate long-term benefits before the widespread adoption of the procedure is recommended.

Limitations of the study include the short follow-up and the fact that outcomes are limited to observation of clinical parameters. None of the dogs died or were euthanized and, therefore, the security of the clamp device on the valves and its effect on the valve leaflets could not be evaluated post-mortem. Furthermore, the dogs included in this study were limited to dogs with mitral regurgitation but no clinical signs and minimal, if any, chamber enlargement (stage B1 MMVD). The efficacy of this device still needs to be further tested on patients with varying stages of MMVD.

Echocardiography is required to perform this procedure. It is difficult to position the clamp precisely with two-dimensional imaging and thus there is a risk that the clamp may not be centered correctly in the mitral valve. Three-dimensional echocardiography would be optimal for this procedure (23). Excessive manipulation of the device during its implantation could result in injuries to the mitral valve leaflets. Valve lesions can rarely be perceived via echocardiography; therefore, we cannot be confident that such a complication did not occur. However, any injury caused was not manifested as a worsening of mitral regurgitation and we expect that this problem will be less of concern with increasing experience and improved handling. The trans-apical access to the left ventricle may result in limited application of this procedure in some patients with left ventricular disorders, which may be resolved through more sophisticated surgical techniques.

To the authors' knowledge, this is the first report of successful use of a transcatheter procedure to surgically manage mitral valve regurgitation in dogs with naturally occurring MMVD.

In conclusion, the edge-to-edge valve closure using the ValveClamp mitral valve system is easy to perform and is highly effective at reducing the severity of mitral regurgitation. The system has good potential to be used in clinical practice on canine MMVD patients, although more data needs to be collected to prove its long-term safety and efficacy.

## Data Availability Statement

The original contributions presented in the study are included in the article/Supplementary Materials, further inquiries can be directed to the corresponding author.

## Ethics Statement

The animal study was reviewed and approved by the animal welfare and ethics committee of China Agricultural University and the State Key Laboratory of Cardiovascular Diseases Ethics Committee. Written informed consent was obtained from the owners for the participation of their animals in this study.

## Author Contributions

BL was the main surgeon of all surgeries. SL and JL designed the study. WP and FZ were the assistant surgeons of all surgeries. LJ and XZ monitored the patients during anesthesia, recorded, and analyzed the data. BL, SL, and JL wrote the manuscript. All authors contributed to the article and approved the submitted version.

## Conflict of Interest

The authors declare that the research was conducted in the absence of any commercial or financial relationships that could be construed as a potential conflict of interest.
